# Shorter sleep duration and better sleep quality are associated with greater tissue density in the brain

**DOI:** 10.1038/s41598-018-24226-0

**Published:** 2018-04-11

**Authors:** Hikaru Takeuchi, Yasuyuki Taki, Rui Nouchi, Ryoichi Yokoyama, Yuka Kotozaki, Seishu Nakagawa, Atsushi Sekiguchi, Kunio Iizuka, Yuki Yamamoto, Sugiko Hanawa, Tsuyoshi Araki, Carlos Makoto Miyauchi, Takamitsu Shinada, Kohei Sakaki, Takayuki Nozawa, Shigeyuki Ikeda, Susumu Yokota, Magistro Daniele, Yuko Sassa, Ryuta Kawashima

**Affiliations:** 1 Division of Developmental Cognitive Neuroscience, Institute of Development, Aging and Cancer, Tohoku University, Sendai, Japan; 2 Division of Medical Neuroimaging Analysis, Department of Community Medical Supports, Tohoku Medical Megabank Organization, Tohoku University, Sendai, Japan; 3 Department of Radiology and Nuclear Medicine, Institute of Development, Aging and Cancer, Tohoku University, Sendai, Japan; 4 Creative Interdisciplinary Research Division, Frontier Research Institute for Interdisciplinary Science, Tohoku University, Sendai, Japan; 5 Human and Social Response Research Division, International Research Institute of Disaster Science, Tohoku University, Sendai, Japan; 6 Smart Ageing International Research Center, Institute of Development, Aging and Cancer, Tohoku University, Sendai, Japan; 7 School of Medicine, Kobe University, Kobe, Japan; 8 Division of Clinical research, Medical-Industry Translational Research Center, Fukushima Medical University School of Medicine, Fukushima, Japan; 9 Department of Functional Brain Imaging, Institute of Development, Aging and Cancer, Tohoku University, Sendai, Japan; 10 Department of Psychiatry, Tohoku Pharmaceutical University, Sendai, Japan; 11 Department of Behavioral Medicine, National Institute of Mental Health, National Center of Neurology and Psychiatry, Tokyo, Japan; 12 Department of Psychiatry, Tohoku University Graduate School of Medicine, Sendai, Japan; 13 ADVANTAGE Risk Management Co., Ltd, Tokyo, Japan; 14 Graduate School of Arts and Sciences, Department of General Systems Studies, The University of Tokyo, Tokyo, Japan; 15 Department of Ubiquitous Sensing, Institute of Development, Aging and Cancer, Tohoku University, Sendai, Japan; 16 School of Electronic, Electrical and Systems Engineering, Loughborough University, England, UK

## Abstract

Poor sleep quality is associated with unfavorable psychological measurements, whereas sleep duration has complex relationships with such measurements. The aim of this study was to identify the associations between microstructural properties of the brain and sleep duration/sleep quality in a young adult. The associations between mean diffusivity (MD), a measure of diffusion tensor imaging (DTI), and sleep duration/sleep quality were investigated in a study cohort of 1201 normal young adults. Positive correlations between sleep duration and MD of widespread areas of the brain, including the prefrontal cortex (PFC) and the dopaminergic systems, were identified. Negative correlations between sleep quality and MD of the widespread areas of the brain, including the PFC and the right hippocampus, were also detected. Lower MD has been previously associated with more neural tissues in the brain. Further, shorter sleep duration was associated with greater persistence and executive functioning (lower Stroop interference), whereas good sleep quality was associated with states and traits relevant to positive affects. These results suggest that bad sleep quality and longer sleep duration were associated with aberrant neurocognitive measurements in the brain in healthy young adults.

## Introduction

Sleep is an essential part of life, and the duration and quality of sleep are associated with many important variables that impact the quality of life. For example, too little or too much sleep is associated with an increased risk for the development of obesity, alcohol dependence, disease progression, and mortality,(for summary, see^1^) as well as memory impairment in the elderly^2^. In children, poor sleep quality and shorter sleep duration are associated with poor academic performance, although this association tends to disappear as children age^3^. Short sleep duration is associated with lower cognitive performance, particularly in early childhood^4^. In addition, extended sleep duration is strongly associated with lower levels of extroversion in the young adult, whereas better sleep quality is associated with many positive affects^1,5^. In addition, interventional studies have shown that sleep deprivation negatively impacts mood and lowers cognitive performance^6^.

Neuroscientific studies have demonstrated the involvement of dopamine in the modulation of sleep and waking (for review of this hypothesis, see^7^). Large doses of dopamine receptor agonists induce behavioral arousal and increased waking^7^, while compounds with dopamine receptor blocking properties reduce waking^7^. Studies of knockout mice also indicated that postsynaptic dopamine D1 and D2 receptors have a facilitatory role in the modulation of behavioral arousal^7^. Further, Parkinson’s disease, which is characterized by the deficiency of dopamine, results in excessive day time sleepiness^7^.

Previous neuroimaging studies investigating conditions related to sleep using volumetry methods revealed that subjects with sleep disorders, which are characterized by impairments to the amount, quality, and timing of sleep, and children who sleep less had a smaller regional gray matter volume (rGMV) in the hippocampus and areas in the prefrontal cortex (PFC), which are involved in the response to stress^8,9^. Also, a previous review suggested that the conditions caused by inadequate sleep result from dysfunction of the PFC^10^. Moreover, a previous intervention study showed that sleep deprivation leads to a decrease in the structural integrity of the white matter^11^. On the other hand, mean diffusivity (MD), a measure of diffusion tensor imaging (DTI)^12^, is used to measure microstructural properties of the brain. As we summarized previously^13^, lower MD reflects greater tissue density, such as the increased presence of unspecific cellular structures (i.e., capillaries, synapses, spines, and macromolecular proteins), properties of myelin, the neuronal membrane, and axons; the shape of neurons or glia; and enhanced tissue organization^12,14^.

Further, recent studies reported that MD in the dopaminergic system (MDDS) is uniquely associated with pathological (Parkinson’s disease, substance abuse), pharmacological (dopamine agonist), and trait differences or cognitive changes related to dopamine^15–19^. Further, among the dopaminergic pathways, the MD of the basal ganglia, such as the caudate, putamen, and globus pallidum have been shown to be consistently related to the traits and states that are supposed to be associated with dopaminergic functions^16,20,21^ including extraversion^22^, which is negatively correlated with sleep duration as described above.

As described, sleep is an essential part of life, and sleep duration and quality are critically associated with important variables such as disease prognosis, academic performance, cognitive performance, and general well-being. However, despite the unique importance of sleep duration and quality, the microstructural basis of the sleep duration and sleep quality remains largely unknown. Therefore, the purpose of this study was to investigate this issue. To this end, we hypothesized that (a) longer sleep duration is associated with lower MD in the hippocampus and the PFC based on the previous studies of volumetry^8,9^, (b) shorter sleep duration is associated with lower MDDS, particularly those of the putamen and globus pallidum due to strong associations between prolonged sleep duration and lower extroversion as well as the robust association between low extroversion and high MDDS^23^, and (c) better sleep quality is associated with lower MD in the hippocampus and the PFC, based on previous studies of volumetry^8,9^.

We also investigated the associations between sleep duration, sleep quality, and various psychological variables to support the understanding of the nature of correlates of sleep duration and sleep quality.

## Methods

### Subjects

The present study, which is a part of an ongoing project to investigate the association between brain imaging, cognitive function, and aging, included 1201 healthy, right-handed individuals (693 men and 508 women) for whom relevant sleep-related measures and diffusion imaging data were collected. The mean age of the subjects was 20.7 years [standard deviation (SD), 1.8; age range: 18–27 years old]. For details of subjects’ information, see Supplemental Methods. For the limitation of this study related to subjects’ characteristics, see Supplemental Discussion. Written informed consent was obtained. This study was approved by the Ethics Committee of Tohoku University. All experiments were in accordance with the declaration of Helsinki.

### Sleep measures

The sleep habit questionnaire^24^ was used to assess sleep duration and quality. To assess sleep duration, the study participants were asked “What is the usual length of your sleep?” Potential answers were (1) less than 4 h, (2) approximately 4 h, (3) approximately 4.5 h, ….(13) approximately 9.5 h, (14) approximately 10 h, and (15) more than 10 h, which were converted into hours (“less than 4 h” and “more than 10 h” were converted into 3.5 h and 10.5 h, respectively). This approach of directly asking the subjects how much they sleep has been used in previous studies, and its validity has been substantiated^1^. To assess sleep quality, the study participants were asked “How would you rate the depth of your usual sleep”. Potential answers were (1) can have a sound sleep, (2) can relatively have a sound sleep, (3) neither, (4) relatively bad, and (5) very bad. “juku-sui” in Japanese are translated into sound sleep here. This term represents sleep depth, continuity, and good quality. The response to this question was used as a continuous variable. Subjective sleep measures showed a stronger relationship with performance measures than objective measurements^3^. Our available psychological data showed greater sleep quality measured by this question is associated with (a) a lower score on the sleep disorder scale of General Health Questionnaire 30^25^ (N = 1196, r = −0.372, p = 1.16 × 10^−40^), which includes questions on insomnia, nocturnal awakening, and bad feeling when one is awake, (b) lower number of awakening at night (r) measured by the same sleep habit inventory^24^ (N = 1201, r = −0.383, p = 2.29*10^−43^) and (c) less experience of insomnia measured by the same sleep habit inventory^24^ (N = 1200, r = −0.306, p = 1.63*10^−27^). These results suggest the validity of the question for assessing sleep quality.

This questionnaire has been used in other Japanese studies^26–28^.

To adjust the effects of the socioeconomic status in analyses of the sleep-related measures, data related to the socioeconomic status were collected in accordance with our previous study and mostly with the standard approach used by the Japanese government for evaluating socioeconomic status. For details, see our previous study^29^.

#### Psychological measures

Neuropsychological tests and questionnaires were administered. The mood status of the preceding 1 week for each subject was measured using the shortened Japanese version^30^ of the Profile of Mood States psychological rating scale^31^. The personality traits of each subject were measured using the Japanese version of the Temperament and Character Inventory^32^. The following basic cognitive functions were also analyzed. Details of these tests are described elsewhere^33,34^. Processing speed was measured using the perception factor of the Tanaka B-type intelligence test (type 3B)^35^. General intelligence was measured using Raven’s advanced progressive matrices (RAPM)^36^. Creativity measured by divergent thinking was measured using the S-A creativity test^37^. Executive function was measured by the stroop interference of matching type Hakoda’s version of Stroop task^38^. Some of these measures were developed in Japanese and used, but the reliability and validity have been shown previously. (For summary, see^39,40^).

### Behavioral data analysis

The behavioral data were analyzed using SPSS 18.0 statistical software (SPSS Inc., Chicago, IL). The descriptions in this subsection were mostly reproduced from our previous study^33^. Sex differences in demographic variables were tested using two-tailed *t*-tests. In each analysis, *P* < 0.05 was considered statistically significant. These were only descriptive analyses, and they were not relevant to the aim of this study; thus, corrections for multiple comparisons were not performed. Associations among demographic variables were analyzed using multiple regression analyses with age and sex as covariates. In these analyses, results with a threshold of *P* < 0.05 were considered to be statistically significant, after correcting for the false discovery rate (FDR) using the graphically sharpened method^41^.

### Image acquisition and analysis

MRI data acquisition was conducted using a 3T Philips Achieva scanner. Diffusion-weighted data were acquired using a spin-echo EPI sequence (TR = 10293 ms, TE = 55 ms, FOV = 22.4 cm, 2 × 2 × 2 mm^3^ voxels, 60 slices, SENSE reduction factor = 2, number of acquisitions = 1). The diffusion weighting was isotropically distributed along 32 directions (*b* value = 1,000 s/mm^2^). Additionally, three images with no diffusion weighting (*b* value = 0 s/mm^2^) (b = 0 images), were acquired, using a spin-echo EPI sequence (TR = 10293 ms, TE = 55 ms, FOV = 22.4 cm, 2 × 2 × 2 mm^3^ voxels, 60 slices). FA and MD maps were calculated from the collected images using a commercially available diffusion tensor analysis package on the MR console. For more details, see Supplemental Methods. Descriptions in this subsection were mostly reproduced from a previous study using similar methods^13^.

#### Preprocessing of imaging data

Preprocessing and analysis of imaging data were performed using SPM8 implemented in Matlab. Basically, we normalized MD images of subjects with previously validated diffeomorphic anatomical registration through exponentiated lie algebra (DARTEL)-based registration process method to give images with 1.5 × 1.5 × 1.5 mm^3^ voxels, then tissues that are not likely to be gray or white matter were carefully removed and smoothed by convolving them with an isotropic Gaussian kernel of 6-mm full width at half maximum. For details, see Supplemental Methods.

#### Whole-brain statistical analysis

We investigated MD associated with individual differences in sleep quality and sleep duration. The statistical analyses of imaging data were performed with SPM8. In these analyses, we performed a whole brain multiple regression analysis. These analyses were performed with sex, age, family annual income, parents’ average highest educational qualifications, total intracranial volume (TIV) that was calculated as described previously^42^, sleep quality, and sleep duration as covariates. The analyses were limited to the gray + white matter mask, which was created as described above.

A multiple comparison correction was performed using threshold-free cluster enhancement (TFCE)^43^ with randomized (5,000 permutations) nonparametric testing using the TFCE toolbox (http://dbm.neuro.uni-jena.de/tfce/). We applied a threshold of FWE corrected at *P* < 0.05.

With a possible concern of the effects of regional tissue probability, we additionally performed voxel-by-voxel whole brain multiple regression analyses that corrected the effects of rGMD and rCSFD. We confirmed that these corrections of rGMD and rCSFD did not substantially alter the extents of the effects of sleep duration and quality in the MD analyses, which are seen in the Results section of the main text. For details, see Supplemental Methods, Supplemental Results, Supplemental Figs 2a–d and 3a,b).

#### Analyses of nonlinear associations between sleep duration and neurocognitive measures

As reported in our previous article^16^, to determine whether the linear or quadratic function was a better fit for the trajectory of sleep duration with dependent neurocognitive measures, the correlations between sleep duration and psychological variables and MD in the regions of interest (ROIs) were estimated using linear and quadratic functions. The best-fit model was determined by selecting the function with the smallest Akaike information criteria (AIC)^44^. Corrections for multiple comparisons were not performed because it is difficult to evaluate these parameters (multiple testing for each neurocognitive measure, correlation between the two models, etc.). The ROIs were the bilateral putamen, globus pallidum, and hippocampus (see Introduction). All ROIs were constructed using the WFU PickAtlas Tool’s aal option (http://www.fmri.wfubmc.edu/cms/software#PickAtlas) and limited to areas strongly likely to be gray or white matter, as described elsewhere^16^.

## Results

### Basic data

The distributions of sleep duration and sleep quality in the present sample are presented in Supplemental Fig. 1. Sleep duration and quality were slightly correlated (simple correlation, r = 0.0851, *p* = 0.003). The average results (and SDs) of age, RAPM score, and sleep duration and quality for the men and women included in our sample are shown in Supplemental Table 1. Two-tailed t-tests showed that males slept significantly longer than females (*p* < 0.001, *t* = 4.511). Sleep quality was not significantly different between males and females. The average sleep duration of 6.87 h in males and 6.59 h in females is comparable to the average sleep duration of 6.44 h in males and 6.32 h in females, respectively, observed in a governmental survey of Japanese adults, although it seems to be changing^45^.

### Linear associations between sleep duration, quality, and psychological variables

Multiple regression analyses were performed with each psychological covariate as a dependent variable and age, sex, socioeconomic status, sleep duration, and sleep quality as independent variables. After correction for multiple comparisons, sleep duration was significantly negatively correlated with personalities of persistence, cooperativeness, and self-transcendence and positively correlated with Stroop interference, indicating that longer sleep duration is associated with lower executive functions. On the other hand, sleep quality was negatively correlated with moods of tension-anxiety, depression-dejection, anger-hostility, fatigue-inertia, confusion-bewilderment, personalities of harm avoidance and positively correlated with mood of vigor-activity, personalities of reward dependence, persistence, self-directedness, and cooperativeness. For statistical values, see Supplemental Table 2.

#### Whole-brain analyses of the correlations between sleep duration, sleep quality, and MD

**Figure 1:**
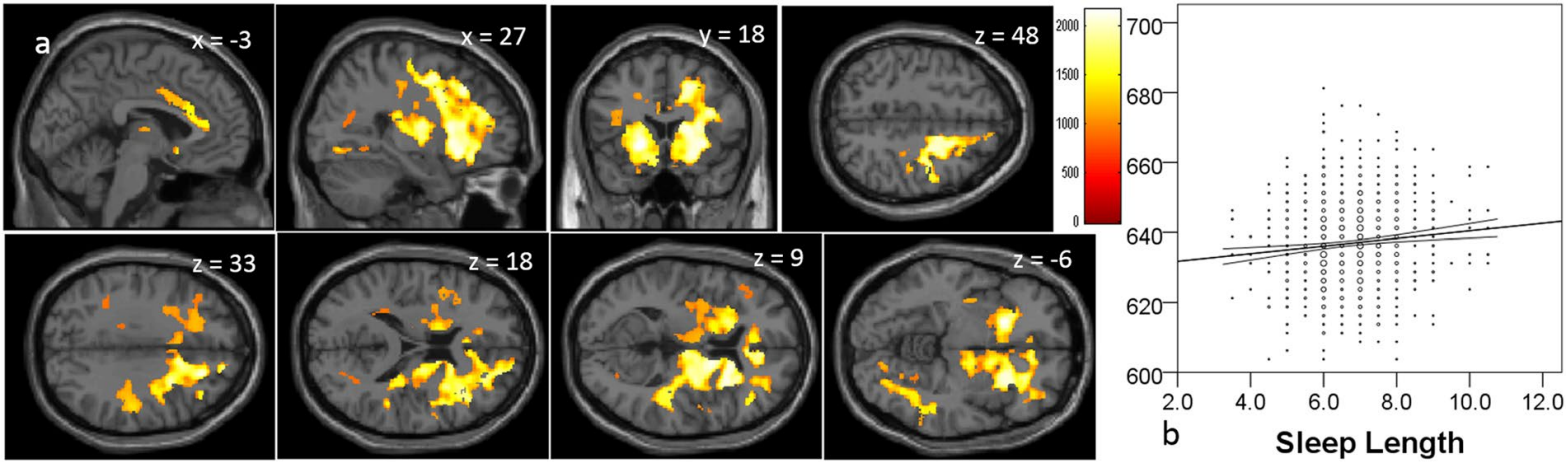
Positive mean diffusivity correlates of the sleep duration (hours). (**a**) The results shown were obtained using a threshold of threshold-free cluster enhancement (TFCE) of *P* < 0.05, based on 5000 permutations. The results were corrected at the whole brain level. Regions with significant correlations are overlaid on a “single subject” T1 image of SPM8. The color represents the strength of the TFCE value. Significant positive correlations with MD were observed in extensive gray and white matter regions that were mainly focused in the PFC, basal ganglia architecture, and anterior part of the corpus callosum. (**b**) A scatter plot with a trend line depicting correlations between mean MD in the largest significant cluster and hours of sleep duration. 95% confidence intervals for the trend lines are also shown.

Whole-brain multiple regression analysis showed that sleep duration was significantly and positively correlated with MD in the huge anatomical cluster that mainly encompassed the PFC, basal ganglia architectures, and anterior part of the corpus callosum (Fig. 1, Supplemental Table 3).

**Figure 2:**
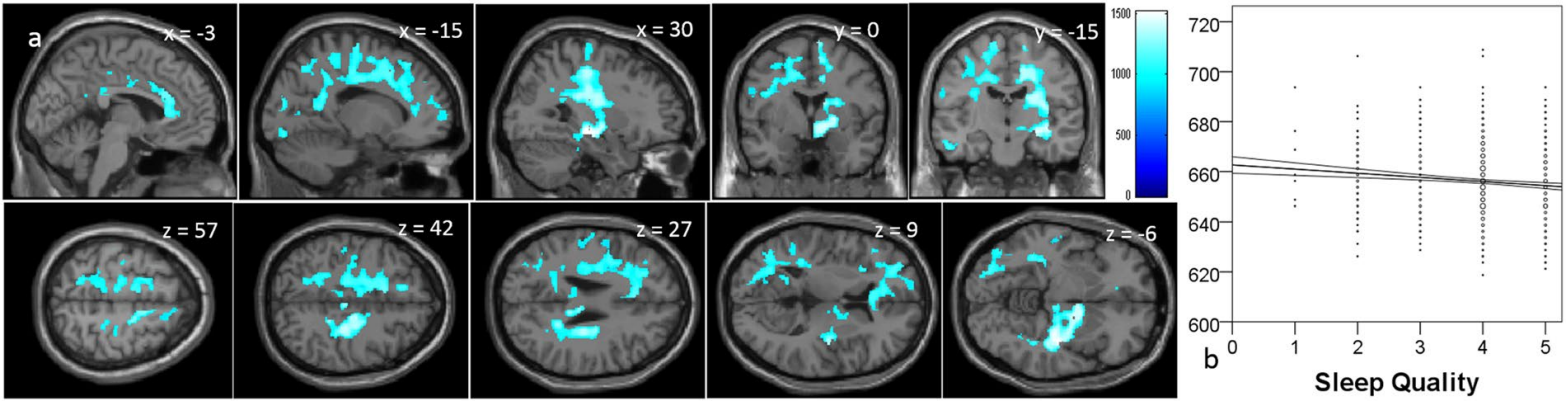
Negative mean diffusivity is correlated with sleep quality. (**a**) The results shown were obtained using a threshold of threshold-free cluster enhancement (TFCE) of *P* < 0.05, based on 5000 permutations. The results were corrected at the whole brain level. Regions with significant correlations are overlaid on a “single subject” T1 image of SPM8. The color represents the strength of the TFCE value. Significant negative correlations with MD were observed in extensive gray and white matter regions across most of areas in the brain, including the right hippocampus (unlike the case of MD correlates of sleep duration), but that did not substantially overlap with basal ganlia architectures (unlike the case of MD correlates of sleep duration). (**b**) A scatter plot with a trend line depicting correlations between mean MD in the largest significant cluster and hours of sleep quality. 95% confidence intervals for the trend lines are also shown.

Whole-brain multiple regression analysis showed that the sleep quality was significantly and negatively correlated with MD in the huge anatomical cluster that encompassed most areas of the brain, including the right hippocampus (unlike the case of MD correlated with sleep duration), but that did not substantially overlap with the basal ganglia architecture (unlike the case of MD correlated with sleep duration) (Fig. 2, Supplemental Table 4).

Unlike in our previous reports, in this study, we did not correct the effects of regional gray matter density and regional CSF density on a voxel-by-voxel basis^46^. This was done to allow application of the abovementioned permutation software using TFCE, because the two programs are incompatible. However, the results correcting for regional gray matter density and regional CSF density on a voxel-by-voxel basis (see our previous study for details of this method)^46^ and results not correcting for these are comparable under the normal false discovery rate threshold of SPM (See Supplemental Methods, Supplemental Results, and Supplemental Fig. 2, 3). Therefore, partial volume effects are of no concern.

### Identification of linear and nonlinear associations between sleep duration and neurocognitive measures

**Figure 3:**
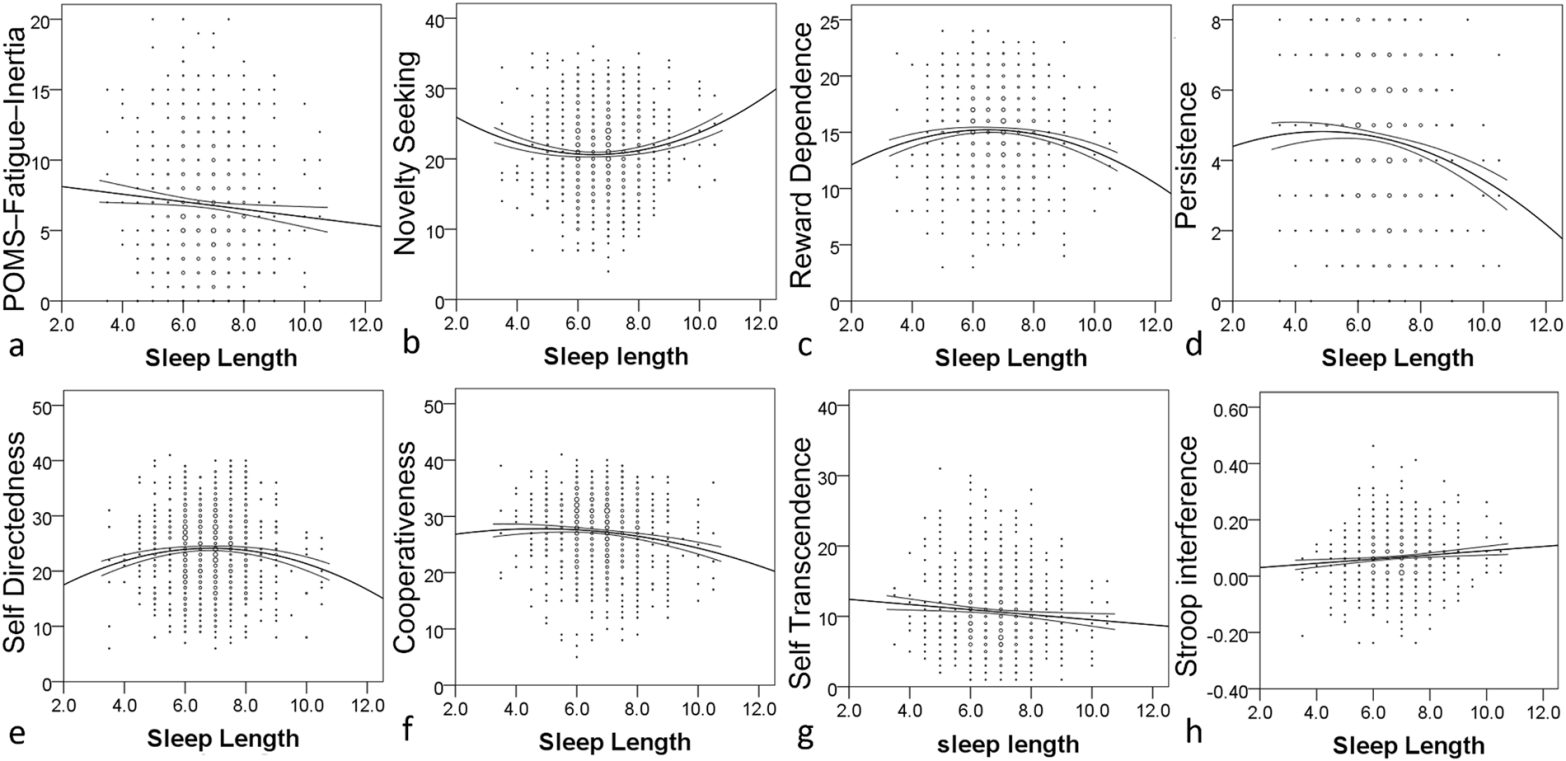
Linear and non-linear associations between sleep duration and psychological variables. Fitted lines and curves as well as their 95% confidence intervals were added. The association of sleep duration with (**a**) fatigue state, (**b**) novelty seeking, (**c**) reward dependence, (**d**) persistence, (**e**) self-directedness, (**f**) cooperativeness, (**g**) self-transcendence, and (**h**) Stroop interference.

Next, considering that too little and too much sleep were associated with some risk factors in previous studies, we determined whether a linear or quadratic function was a better fit for the association between sleep duration and psychological variables as well as MD in the ROIs. When the *p* value of the best fit model was less than 0.05, sleep duration showed a quadratic positive correlation with novelty seeking; a quadratic negative correlation with reward dependence, persistence, self-directedness, and cooperativeness; a linear negative correlation with self-transcendence and fatigue state score; and a linear positive correlation with Stroop interference. Sleep duration was not associated with MD in the bilateral hippocampus but showed a near significant tendency of a linear positive association with MD in the bilateral globus pallidum and a significant linear positive correlation with the left putamen. Although, the best fit significant model of the associations between sleep duration and right putamen was quadratic positive, the difference in AIC between the linear and quadratic associations were minimal; therefore, robust evidence that quadratic functions in the associations between MD and sleep duration was not obtained. For the actual trajectory, see Fig. 3 and for statistical values, see Supplemental Table 5. No other significant associations were observed.

Further, sleep duration and sleep quality did not exhibit quadratic association. Given the abovementioned slight linear correlation between sleep duration and sleep quality, disentangling the two effects is not necessary.

## Discussion

In this study, associations between MD, psychological measures, and sleep duration/quality were investigated. Our novel findings showed that shorter sleep duration and better sleep quality were associated with lower MD (which is generally associated with more neural tissues) in widespread areas of the brain. Particularly, consistent with one of our hypotheses, sleep duration was positively associated with MDDS, while the association between sleep quality and MDDS was minor, and MD in the right hippocampus was negatively associated with sleep quality, unlike sleep duration. But contrary to our hypothesis, sleep duration was positively associated with MDDS and widespread regions of the brain, including the PFC. Further, while sleep duration was associated with several personality traits in a quite complex manner and better sleep quality was associated with better mood states, a shorter sleep duration was associated with better executive functioning (lower Stroop interference).

Our findings suggested that in healthy young adults, longer sleep duration and poorer sleep quality is indicative of aberrant neural conditions. In this study, sleep duration was linearly and positively associated with MD in the widespread areas of the brain (Fig. 1). As described in the Introduction, a smaller MD is supposedly associated with more neural tissues, which prohibit free diffusion of water molecules with smaller cellular structures (e.g., capillaries, synapses, spines, and macromolecular proteins); properties of myelin, neuronal membranes, and axons; the shape of neurons and glia; and enhanced tissue organization^12,14^. In addition, our previous studies of large samples showed that a greater MD in the widespread areas of the brain, including the prefrontal areas, is associated with lower performance IQ in children^13^, as well as the existence of the genotype of the dopamine receptor D4 gene, which is associated with attention deficit hyperactive disorder^46^. However, it is difficult to completely conclude that a greater MD is associated with aberrant neural mechanisms. However, in the present study, shorter sleep duration was associated with better executive functions (less Stroop interference). Also, although sleep duration is associated with personalities in a complex manner, a shorter sleep duration is associated with greater persistence, while subjects with greater persistence are industrious, hard-working, persistent, and stable despite frustration. Considering these, greater MD in subjects with longer sleep duration may be indicative of aberrant neural mechanisms. Similarly, less MD was associated with better sleep quality in widespread areas of the brain. Consistently, better sleep quality was associated with better mood states as well as greater persistence and less personalities with negative affect (harm avoidance).

The present results of the sleep duration in the areas of the dopaminergic system are congruent with the notion of the involvement of dopamine in the modulation of sleep and wake cycles that was described in Introduction. In the present study, we hypothesized that sleep duration was positively correlated with MDDS. Previously, we have shown that a decreased MDDS is associated with states that are shown to be associated with facilitated dopaminergic function, such as a motivational state)^20^, and traits that are suggested to be associated with facilitated dopaminergic function, such as extraversion^23^, novelty seeking, and persistence^16^. Together with a wide range of evidence, it has been hypothesized that a lower MDDS is associated with conditions related to facilitated dopaminergic functions^47^. Therefore, our results of the association of shorter sleep duration with lower MDDS is consistent with animal, physiological, and pathological studies that have suggested that facilitated dopaminergic functions generally tend to be associated with an increased behavioral arousal and waking^7^.

The cross-sectional macro-level neuroimaging studies were insufficient to reveal the causal relationship and physiological mechanisms that underlie the observed associations. Therefore, we did not attempt to identify these mechanisms in this study. However, for reference, we suggest a few possible mechanisms. First, sleep duration was associated with several traits in quite complex manners (Fig. 3). Considering that traits are not easily changed by definition, sleep duration may be determined by the personalities or neural mechanisms that are associated with such traits. For example, high-dose administration of agonists of dopamine receptors D1 and D2 reduces all types of sleep^7^. In addition, these receptors are most pronouncedly expressed in the putamen^48^. On the other hand, personality is likely to be driven by augmentation of dopamine function, and motivation (persistence) is robustly associated with lower MDDS^16^. In consideration of these findings, structures of the dopaminergic system may underlie MDDS, less sleep duration, and persistence. D1 receptors are expressed in the basal ganglia architecture, as well as widespread areas in the cortex, which might explain the positive associations between sleep duration and MD, which was contrary to our hypothesis. On the other hand, poor sleep quality is physiologically considered or sometimes defined as a condition in which deep sleep or the deeper stages of non-rapid eye movement sleep, i.e., stages 3 and 4, also known as slow-wave sleep, is prohibited^49^. In addition, this sleep is considered to be the most restorative part of sleep, and this sleep stage is critical for neural plasticity processes and memory consolidation for summary, see^49,50^. Further, unlike sleep duration, the results of the present study showed that sleep quality was mostly associated with mood states. In consideration of these findings, poor sleep quality may lead to worse mood states due to prohibited restorative stages, and poor sleep quality may lead to high MD (less neural tissues) due to prohibited processes of neural plasticity, particularly those in the hippocampus that play critical roles in memory consolidation^51^. However, this is pure speculation; thus, future human and animal studies are needed to investigate how sleep deprivation and intervention lowers sleep quality as well as how genetic differences of relevant dopamine-related functional polymorphisms can change micro-level neural mechanisms and MD.

The findings of this study will advance the current understanding of the neural correlates of sleep duration and sleep quality. With respect to sleep quality, as described in the Introduction, poor sleep quality is associated with negative affect^5^ and poor academic performance^3^, and conditions that are characterized by poor sleep quality have been associated with reduced rGMV in the areas of the PFC and hippocampus^9^. Our findings of greater MD in subjects with poor sleep quality are in agreement with those of previous studies, and conditions associated with poor sleep quality are undesirable. On the other hand, as described in the Introduction, short sleep duration has been previously associated with poor academic and cognitive performance^3^ and reductions in the hippocampus and rGMV in the areas of the PFC in children^8^. Moreover, interventional studies have shown that sleep deprivation lowers moods and cognitive performance^6^. In addition, an intervention study has shown that sleep deprivation leads to a decrease in the structural integrity of the white matter and a measure of diffusivity^11^. So, overall expectation from these previous studies would be that conditions in which subjects are not sleeping much are mostly aberrant in the present sample, too. However, in this sample of normal young adults, shorter sleep duration was associated with better executive functioning, higher persistence, and lower MD in widespread areas of the brain, despite a state of increased fatigue. These results suggest that the condition that the sleep was deprived experimentarily and the condition in subjects are not sleeping much in the real life are substantially different, and the latter should not be treated as some aberrant condition in the normal young adult.

In conclusion, better sleep quality and shorter sleep duration were associated with lower MD in healthy young adults. In addition, shorter sleep duration was associated with better executive functioning. Contrary to the indications reported in previous studies, shorter sleep duration is not reflective of some unfavorable neurocognitive mechanisms in healthy young adults.

## Electronic supplementary material

Supplementary online material

## References

[1] Vincent N, Cox B, Clara I (2009). Are personality dimensions associated with sleep length in a large nationally representative sample?. Compr Psychiatry.

[2] Xu L (2011). Short or long sleep duration is associated with memory impairment in older Chinese: the Guangzhou Biobank Cohort Study. Sleep.

[3] Dewald JF, Meijer AM, Oort FJ, Kerkhof GA, Bögels SM (2010). The influence of sleep quality, sleep duration and sleepiness on school performance in children and adolescents: a meta-analytic review. Sleep medicine reviews.

[4] Touchette É (2007). Associations between sleep duration patterns and behavioral/cognitive functioning at school entry. Sleep.

[5] Gray EK, Watson D (2002). General and specific traits of personality and their relation to sleep and academic performance. J Pers.

[6] Pilcher JJ, Huffcutt AJ (1996). Effects of sleep deprivation on performance: a meta-analysis. Sleep: Journal of Sleep Research & Sleep Medicine.

[7] Monti JM, Monti D (2007). The involvement of dopamine in the modulation of sleep and waking. Sleep medicine reviews.

[8] Taki Y (2011). Sleep duration during weekdays affects hippocampal gray matter volume in healthy children. Neuroimage.

[9] Weng H-H (2014). Mapping gray matter reductions in obstructive sleep apnea: an activation likelihood estimation meta-analysis. Sleep.

[10] Dahl RE (1996). The impact of inadequate sleep on children’s daytime cognitive function. Semin Pediatr Neurol.

[11] Elvsåshagen T (2015). Widespread Changes in White Matter Microstructure after a Day of Waking and Sleep Deprivation. PLoS ONE.

[12] Beaulieu C (2002). The basis of anisotropic water diffusion in the nervous system–a technical review. NMR Biomed.

[13] Takeuchi H (2016). Impact of videogame play on the brain’s microstructural properties: Cross-sectional and longitudinal analyses. Mol Psychiatry.

[14] Sagi Y (2012). Learning in the fast lane: new insights into neuroplasticity. Neuron.

[15] Takeuchi H (2015). Working memory training impacts the mean diffusivity in the dopaminergic system. Brain Struct Funct.

[16] Takeuchi H (2015). Mean diffusivity of globus pallidus associated with verbal creativity measured by divergent thinking and creativity-related temperaments in young healthy adults. Hum Brain Mapp.

[17] Razek AA, Elmongy A, Hazem M, Zakareyia S, Gabr W (2011). Idiopathic Parkinson disease effect of levodopa on apparent diffusion coefficient value of the brain. Acad Radiol.

[18] Péran P (2010). Magnetic resonance imaging markers of Parkinson’s disease nigrostriatal signature. Brain.

[19] Alicata D, Chang L, Cloak C, Abe K, Ernst T (2009). Higher diffusion in striatum and lower fractional anisotropy in white matter of methamphetamine users. Psychiatry Research: Neuroimaging.

[20] Takeuchi, H. *et al*. Mean diffusivity of basal ganglia and thalamus specifically associated with motivational states among mood states. *Brain Struct Funct* Epub ahead of publication (2016). 10.1007/s00429-016-1262-5 27364694

[21] Nakagawa, S. *et al*. Basal ganglia correlates of fatigue in young adults. *Scientific reports* **6**, article 21386 (2016). 10.1038/srep21386 PMC4759547 26893077

[22] Yokoyama R (2014). Association between gray matter volume in the caudate nucleus and financial extravagance: findings from voxel-based morphometry. Neurosci Lett.

[23] Association between extroversion and regional mean diffusivity. *Proceedings of the Physiological seminar of Heisei 26 fiscal year* Okazaki, Japan (2014).

[24] Miyashita, A. Sleep habit inventory (life habit inventory). In: Research TJSoS (ed). *Handbook of Sleep Science and Sleep Medicine*. Asakura-syoten: Tokyo, pp 533–538 (1994).

[25] Iwata N, Uno B, Suzuki T (1994). Psychometric Properties of the 30‐item Version General Health Questionnaire in Japanese. Psychiatry Clin Neurosci.

[26] Oda S, Seino A, Moriya K (2001). Survey on the relation between the quality of nocturnal sleep and habitual exercise among young university students. Japanese Journal of Physical Fitness and Sports Medicine.

[27] Nakamura M (2004). On Psychosomatic Symptoms and Sleep Disturbances of University Students [in Japanese]. Journal of clinical and educational psychology.

[28] Yamakawa K, Mizuta T, Fujisawa K, Ohira H (2008). Assessment of sleep timecourse of Good Sleeper and Poor Sleeper. Japanese journal of Human Environmental Research.

[29] Takeuchi H (2014). Anatomical correlates of quality of life: Evidence from voxel-based morphometry. Hum Brain Mapp.

[30] Yokoyama, K. *POMS Shortened Version (in Japanese)*. Kanekoshobo: Tokyo (2005).

[31] McNair, D. M., Lorr, M., Droppleman, L. F. *Profile of mood states*. Educational and Industrial Testing Service: San Diego, California (1992).

[32] Kijima N (1996). Cloninger-no-kishitsu-to-seikaku-no-7inshimodel-oyobi-nihongoban [Cloninger’s seven-factor model of temperament and character and Japanese version of Temperament and Character Inventory (TCI)]. Seishinka-shindangaku [Archives of Psychiatric Diagnosis and Clinical Evaluation].

[33] Takeuchi H (2015). Regional gray matter density is associated with morningness–eveningness: Evidence from voxel-based morphometry. Neuroimage.

[34] Takeuchi H (2015). Degree centrality and fractional amplitude of low-frequency oscillations associated with Stroop interference. Neuroimage.

[35] Tanaka, K, Okamoto, K, Tanaka, H. *Manual of New Tanaka B type intelligence test*. Kaneko Syobo: Tokyo (2003).

[36] Raven, J. *Manual for Raven’s progressive matrices and vocabulary scales*. Oxford Psychologists Press: Oxford (1998).

[37] Society_For_Creative_Minds. *Manual of S-A creativity test*. Tokyo shinri Corporation: Tokyo, Japan (1969).

[38] Hakoda Y, Sasaki M (1990). Group version of the Stroop and reverse-Stroop Test: The effects of reaction mode, order and practice. Kyoikushinrigakukenkyu (Educational Psychology Research).

[39] Takeuchi, H, *et al*. Association of hair iron levels with creativity and psychological variables related to creativity. *Frontiers in Human Neuroscience* **7**, **Article875**, 1–9 (2013). 10.3389/fnhum.2013.00875 PMC3866515 24385960

[40] Takeuchi H (2012). Regional gray and white matter volume associated with Stroop interference: Evidence from voxel-based morphometry. Neuroimage.

[41] Benjamini Y, Hochberg Y (2000). On the adaptive control of the false discovery rate in multiple testing with independent statistics. Journal of Educational and Behavioral Statistics.

[42] Hashimoto T (2015). Neuroanatomical correlates of the sense of control: Gray and white matter volumes associated with an internal locus of control. Neuroimage.

[43] Smith SM, Nichols TE (2009). Threshold-free cluster enhancement: addressing problems of smoothing, threshold dependence and localisation in cluster inference. NeuroImage.

[44] Akaike H (1974). A new look at the statistical model identification. Automatic Control, IEEE Transactions on.

[45] Japan_Ministry_of_Health_Labor_Welfare. Annual report of the national health and nutrition survey in 2015 (in Japanese). Tokyo (2016).

[46] Takeuchi H (2015). Cognitive and neural correlates of the 5-repeat allele of the dopamine D4 receptor gene in a population lacking the 7-repeat allele. Neuroimage.

[47] Takeuchi, H. & Kawashima, R. Mean diffusivity in the dopaminergic system and neural differences related to dopaminergic system. *Current neuropharmacology* in press. 10.2174/1570159X15666171109124839 PMC6018195 29119929

[48] McNab F (2009). Changes in Cortical Dopamine D1 Receptor Binding Associated with Cognitive Training. Science.

[49] Tasali E, Leproult R, Ehrmann DA, Van Cauter E (2008). Slow-wave sleep and the risk of type 2 diabetes in humans. Proceedings of the National Academy of Sciences.

[50] Walker MP, Stickgold R (2004). Sleep-dependent learning and memory consolidation. Neuron.

[51] Nadel L, Moscovitch M (1997). Memory consolidation, retrograde amnesia and the hippocampal complex. Curr Opin Neurobiol.

