# Supplementary material for: Shorter sleep duration and better sleep quality are associated with greater tissue density in the brain
Source: Sci Rep. 2018 Apr 11;8:5833. doi: 10.1038/s41598-018-24226-0 (PMC5895621; doi:10.1038/s41598-018-24226-0)
Supplement: Supplementary file 1 — Supplementary online material [file 41598_2018_24226_MOESM1_ESM.pdf]

Shorter sleep duration and better sleep quality are associated with greater tissue density in the brain

Hikaru Takeuchi[a], Yasuyuki Taki[a,b,c], Rui Nouchi[d,e,f], Ryoichi Yokoyama[g], Yuka Kotozaki[h], Seishu Nakagawa[i,j], Atsushi Sekiguchi[b,i,k], Kunio Iizuka[l], Yuki Yamamoto[i], Sugiko Hanawa[i], Tsuyoshi Araki[m], Carlos Makoto Miyauchi[n], Takamitsu Shinada[i], Kohei Sakaki[i], Takayuki Nozawa[o], Shigeyuki Ikeda[o], Susumu Yokota[a], Magistro Daniele[p], Yuko Sassa[a], Ryuta Kawashima[a,f,i]

[a]*Division of Developmental Cognitive Neuroscience, Institute of Development, Aging and Cancer, Tohoku University, Sendai, Japan*

[b]*Division of Medical Neuroimaging Analysis, Department of Community Medical Supports, Tohoku Medical Megabank Organization, Tohoku University, Sendai, Japan*

[c]*Department of Radiology and Nuclear Medicine, Institute of Development, Aging and Cancer, Tohoku University, Sendai, Japan*

[d]*Creative Interdisciplinary Research Division, Frontier Research Institute for Interdisciplinary Science, Tohoku University, Sendai, Japan*

[e]*Human and Social Response Research Division, International Research Institute of Disaster Science, Tohoku University, Sendai, Japan*

[f]*Smart Ageing International Research Center, Institute of Development, Aging and Cancer, Tohoku University, Sendai, Japan*

[g] *School of Medicine, Kobe University, Kobe, Japan*

[h]*Division of Clinical research, Medical-Industry Translational Research Center, Fukushima Medical University School of Medicine, Fukushima, Japan*

[i]*Department of Functional Brain Imaging, Institute of Development, Aging and Cancer, Tohoku University, Sendai, Japan*

[j]*Department of Psychiatry, Tohoku Pharmaceutical University, Sendai, Japan*

[k]*Department of Adult Mental Health, National Institute of Mental Health, National*

Center of Neurology and Psychiatry, Tokyo, Japan

[l]*Department of Psychiatry, Tohoku University Graduate School of Medicine, Sendai, Japan*

[m]*ADVANTAGE Risk Management Co., Ltd.*

[n]*Graduate School of Arts and Sciences, Department of General Systems Studies, The University of Tokyo, Tokyo, Japan*

[o]*Department of Ubiquitous Sensing, Institute of Development, Aging and Cancer, Tohoku University, Sendai, Japan*

[p]*School of Electronic, Electrical and Systems Engineering, Loughborough University, England*

Corresponding author:

Hikaru Takeuchi

Division of Developmental Cognitive Neuroscience, IDAC, Tohoku University

4-1 Seiryo-cho, Aoba-ku, Sendai 980-8575, Japan

Tel/Fax: +81-22-717-7988

E-mail: takehi@idac.tohoku.ac.jp

Short title: Sleep and mean diffusivity

Keywords: sleep duration, sleep quality, mean diffusivity, diffusion tensor imaging, basal ganglia, prefrontal cortex, hippocampus

Supplemental Methods

Subjects. The present study, which is a part of an ongoing project to investigate the association between brain imaging, cognitive function, and aging, included 1201 healthy, right-handed individuals (693 men and 508 women) for whom relevant sleep-related measures and diffusion imaging data were collected. The mean (± standard deviation, SD) age of the subjects was 20.7 ± 1.8 years (age range, 18–27 years). The following descriptions were mostly reproduced from another study of ours from the same project using the exactly same methods regarding these issues [1]. Some of the subjects who took part in this study also became subjects of our intervention studies (psychological data and imaging data recorded before the intervention were used in this study)[2]. Psychological tests and MRI scans not described in this study were performed together with those described in this study. All subjects were university students, postgraduates, or university graduates of less than one year's standing. All subjects had normal vision and none had a history of neurological or psychiatric illness. Handedness was evaluated using the Edinburgh Handedness Inventory [3]. Written informed consent was obtained from each subject. For nonadult subjects, written informed consent was obtained from their parents (guardians). This study was approved by the Ethics Committee of Tohoku University.

Subjects were instructed to get sufficient sleep, maintain their conditions, eat sufficient breakfast, and to consume their normal amounts of caffeinated foods and drinks in the day of cognitive tests and MRI scans. In addition, subjects were instructed to avoid alcohol the night before the assessment.

Details of diffusion image acquisition. There are acquisitions for phase correction and for signal stabilization and these are not used as reconstructed images. MD and FA maps were calculated from the collected images using a commercially available diffusion tensor analysis package on the MR consol. This practice has been used in many of our previous studies [4-8]. Furthermore, the results of analyses using these image-generated results were congruent with those of previous studies in which other methods were used [9, 10], suggesting the validity of this method. These procedures involved correction for motion and distortion caused by eddy currents. Calculations were performed according to a previously proposed method [11].

Preprocessing of imaging data

Preprocessing and analysis of functional activation data were performed using SPM8 implemented in Matlab. Most of the following descriptions were reproduced from our previous study using the similar methods [12]. First, the skull in the mean b = 0 image of each participant was stripped as described previously [7]; using the resulting image, diffusion images were linearly aligned to the skull-stripped b = 0 image template created previously [7] to assist with the following procedures.

Subsequently, using a previously validated two-step new segmentation algorithm of diffusion images and the previously validated diffeomorphic anatomical registration through exponentiated lie algebra (DARTEL)-based registration process that utilized the information of the FA signal distribution within the white matter tissue for details, see [5], all images, including gray matter segments [regional gray matter density (rGMD) map], white matter segments [regional white matter density (rWMD) map], and cerebrospinal fluid (CSF) segments [regional CSF density (rCSFD) map] of

diffusion images, were normalized. The voxel size of these normalized images was 1.5 × 1.5 × 1.5 mm^3. In these processes, we used the template for the DARTEL process that we created in our previous study from subjects that participated in the same project for details, see [5].

Next, we created average images of normalized rGMD and rWMD images of all subjects whose diffusion imaging data were obtained in the pre-experiment. Subsequently, for the analyses of MD images from the normalized images of the (a) MD, (b) rGMD, and (c) rCSFD maps, we created images where areas that were not strongly likely to be gray or white matter in our averaged normalized rGMD and rWMD images (defined by "gray matter tissue probability + white matter tissue probability < 0.99") were removed (to exclude the strong effects of CSF on MD throughout analyses). These images were then smoothed (6mm full-width half-maximum) and carried through to the second-level analyses of MD.

We did not use T1 weighted structural images for normalization and calculation of GMC and WMC maps for correction. This is because T1 weighted structural images and EPI images have apparent differences due to the distortion caused by 3T MRI and simply it is apparently not suited for the accurate and precise segmentation and normalization images of MD maps.

Models of whole-brain multiple regression analyses for investigating the effects of adjustment on individual differences of rGMD and rCSFD

In addition to the main whole brain analyses in the main text, we performed multimodality voxel-wise multiple regression analyses that adjusted for the effects of rGMD and rCSFD to investigate if the associations between MD and sleep variables are

substantially affected by individual differences of gray matter extent and CSF extent (in analyzed areas, usually rGMD + rCSFD + rWMD = 1, therefore, the inclusion of rGMD and rCSFD is enough). To perform these analyses, we used the biological parametric mapping toolbox of SPM8 [13]. We performed a voxel-by-voxel whole-brain multiple regression analyses. In all these analyses, the dependent variable at each voxel was the MD value at that voxel, and the independent variables included the rGMD and rCSFD values at that voxel, as well as images, sex, age, family's annual income, parents' average highest educational qualifications, total intracranial volume (TIV) that was calculated as described previously [14], sleep quality, and sleep duration. Then, we compared the extent of the effects by using a threshold of $p < 0.001$ uncorrected. Of note, the method of correction of multiple comparisons using TFCE based permutations cannot be used under the biological parametric mapping toolbox.

Supplemental Results

Comparison of results of whole-brain analyses of the correlations between sleep duration, sleep quality, and MD between when rGMD and rCSFD are not corrected and when rGMD and rCSFD are corrected.

When rGMD and rCSFD at each voxel are not corrected (the same analysis design as in the main text), a whole-brain multiple regression analysis showed that sleep duration was positively correlated with MD in the widespread areas of 9969 voxels with a threshold of $p < 0.001$ uncorrected (Supplemental Fig. 2a). When rGMD and rCSFD are corrected at each voxel, a whole brain multiple regression analysis showed that sleep duration was positively correlated with MD in the widespread areas of 9064 voxels with a threshold of $p < 0.001$ uncorrected (Supplemental Fig. 2b). Therefore,

when GMD and rCSFD are corrected at each voxel, the effects became a bit weaker. However, even with a threshold of $p < 0.05$, corrected for false discovery rate (FDR), the former analysis showed significant results in 26278 voxels (Supplemental Fig. 2c) and the latter analysis showed significant results in 23250 voxels with the effects remaining strong enough (Supplemental Fig. 2d).

When rGMD and rCSFD at each voxel are not corrected (the same analysis design as in the main text), a whole-brain multiple regression analysis showed that sleep quality was negatively correlated with MD in the widespread areas of 2998 voxels with a threshold of $p < 0.001$ uncorrected (Supplemental Fig. 3a). When rGMD and rCSFD are corrected at each voxel, a whole brain multiple regression analysis showed that sleep duration was positively correlated with MD in the widespread areas of 4318 voxels with a threshold of $p < 0.001$ uncorrected. Therefore, when GMD and rCSFD are corrected at each voxel, the effects became a bit stronger (Supplemental Fig. 3b).

Supplemental Discussion

Limitations of this study

The present study had at least one other limitation, i.e., limited sampling. Subjects in this study were young and healthy, consisting of mostly undergraduate and postgraduate students. Such limited sampling is a common hazard of studies using college students [15]. With respect to the purpose of this study, shorter sleep duration was shown to negatively impact academic performance in children [16]. Also, primary illnesses lead to changes in sleep duration for summary, see [17]. In addition, Japanese university students are known to spend less time studying, and the Japanese university period is known to be characterized by moratorium [18]. Therefore, compared with

middle-aged working samples, the sleep duration of university students may be less affected by external duties and may be more influenced by internal factors (such as traits and genetic relevant factors). Considering these, the associations between sleep duration and MD may well be different in another group. However, these observations are all the more reason why it was important to focus on the present sample. Nonetheless, future studies are warranted to elucidate the effects of age and baseline illnesses on the association between MD and sleep duration.

Supplemental Table 1.

Demographic variables of the study participants

	Male (N = 693)		Female (N = 508)	
Measure	Mean	SD	Mean	SD
Age	20.81	1.89	20.60	1.60
RAPM	28.77	3.87	28.10	3.80
Sleep duration	6.87	1.10	6.59	1.07
Sleep quality	3.87	0.95	3.96	0.95

Supplemental Table 2.

Statistical results (beta value, *t*-value, uncorrected *p*-values, *p*-value corrected for FDR[a]) for the multiple regression analyses performed using the psychological variables and the covariates of age, sex, parents' average highest educational level, sleep duration, and sleep quality as dependent variables.

Dependent variables		Sleep duration				Sleep quality			
	N	β	*t*	*p* (*uncorrected*)	*p (FDR)*	β	*t*	*p (uncorrected)*	*p (FDR)*
POMS[b]–Tension–Anxiety	1184	-0.003	-0.086	0.932	0.593	-0.097	-3.350	$8.35*10^{-4}$	0.002
POMS–Depression–Dejection	1184	0.022	0.747	0.455	0.382	-0.119	-4.119	$4.07*10^{-5}$	$2.14*10^{-4}$
POMS–Anger–Hostility	1184	0.008	0.287	0.774	0.525	-0.074	-2.549	0.011	0.019
POMS–Vigor–Activity	1184	0.005	0.181	0.856	0.562	0.109	3.738	$1.95*10^{-4}$	0.001
POMS–Fatigue–Inertia	1184	-0.058	-1.985	0.047	0.058	-0.101	-3.473	$5.33*10^{-4}$	0.002
POMS–Confusion–Bewilderment	1184	-0.028	-0.940	0.347	0.317	-0.068	-2.331	0.020	0.029
TCI[c]-Novelty Seeking	1199	0.029	0.978	0.328	0.317	0.018	0.605	0.545	0.382
TCI-Harm Avoidance	1199	0.033	1.185	0.236	0.261	-0.207	-7.403	$2.50*10^{-13}$	$5.25*10^{-12}$

TCI-Reward Dependence	1199	-0.018	-0.637	0.524	0.382	0.065	2.312	0.021	0.029
TCI-Persistence	1199	-0.130	-4.471	$8.51*10^{-6}$	$5.96*10^{-5*}$	0.085	2.962	0.003	0.006
TCI-Self Directedness	1199	-0.019	-0.671	0.502	0.382	0.177	6.260	$5.35*10^{-10}$	$5.62*10^{-9}$
TCI-Cooperativeness	1199	-0.096	-3.362	$7.97*10^{-4}$	0.002*	0.105	3.688	$2.36*10^{-4}$	$825*10^{-4}$
TCI-Self Transcendence	1199	-0.070	-2.398	0.017	0.027*	0.058	2.008	0.045	0.058
RAPM[d]	1201	0.001	0.035	0.972	0.6	0.026	0.903	0.367	0.321
Stroop interference	1196	0.087	2.988	0.003	0.006*	-0.018	-0.624	0.533	0.382
S-A creativity test	1201	0.028	0.949	0.343	0.317	0.018	0.613	0.540	0.382
TBIT[e] percepion factor	1078	0.031	1.026	0.305	0.317	0.054	1.769	0.077	0.09

[a]False discovery rate. [b]Profile of mood scale. [c]Temperament and Character Inventory, [d]Raven's advanced progressive matrices (a general intelligence task). [e]Tanaka B-type intelligence test. [e]

Supplemental Table 3

Brain regions that exhibited significant positive correlations between sleep duration and MD

Included gray matter areas*(number of significant voxels in left and right side of each anatomical area)	Included large bundles** (number of significant voxels in left and right side of each anatomical area)	x	y	z	TFCE value	Corrected *p* value (FWE)	Cluster size (voxel)
Caudate (L:929, R:1445)/Anterior cingulum (L:602, R:1043)/Middle cingulum (L:170, R:456)/Inferior frontal operculum (L:430, R:392)/Inferior frontal orbital area (L:324, R:499)/Inferior frontal triangular (L:268, R:789)/Middle frontal medial area (L:88, R:432)/Middle frontal orbital area (L:47, R:126)/Middle frontal other areas (L:224, R:1610)/Superior frontal medial area (L:22, R:374)/Superior frontal orbital area (L:289, R:445)/Superior frontal other areas (L:251, R:2113)/Fusiform	Genu of corpus callosum (309)/Body of corpus callosum (116)/Cerebral peduncle (R:150)/Anterior limb of internal capsule (L:735, R:919)/Posterior limb of internal capsule (L:231, R:1138)/Retrolenticular part of internal capsule (R:189)/Anterior corona radiata (L:436, R:1266)/Superior corona radiata (L:99, R:651)/Posterior	-18	18	-4.5	2168.18	0.001	40242

gyrus (R:554)/Heschl gyrus (L:1, R:34)/Hippocampus (R:2)/Insula (L:894, R:426)/Lingual gyrus (R:239)/Inferior occipital lobe (R:318)/Pallidum (L:126, R:554)/Paracentral lobule (R:4)/Postcentral gyrus (R:439)/Precentral gyrus (L:68, R:1155)/Putamen (L:1292, R:1454)/Rectus gyrus (L:596, R:761)/Rolandic operculum (L:20, R:66)/Supplemental motor area (R:227)/Supramarginal gyrus (R:105)/Inferior temporal gyrus (R:255)/Middle temporal gyrus (R:244)/Superior temporal gyrus (L:20, R:498)/Thalamus (L:536, R:1222)/Cerebellum (R:20)	corona radiata (R:72)/Posterior thalamic radiation (R:67)/Sagittal stratum (R:10)/External capsule (L:798, R:745)/Cingulum (L:85, R:244)/Superior longitudinal fasciculus (L:91, R:576)/Superior fronto-occipital fasciculus (L:25, R:64)/Inferior fronto-occipital fasciculus (L:221, R:252)/						
Inferior frontal triangular (L:151)	None	-43.5	33	12	950.49	0.038	151
Fusiform gyrus (R:3)/Lingual gyrus (R:42)/	None	25.5	-55.5	-7.5	927.15	0.040	43
Middle frontal orbital area (R:1)/	None	33	60	-7.5	922.79	0.042	1

Calcarine Cortex (R:82)/Cuneus (R:2)/Superior occipital lobe (R:20)/	Posterior corona radiata (R:17)/Posterior thalamic radiation (R:46)/	24	-72	13.5	918.41	0.043	224
None	Splenium of corpus callosum (49)/Retrolenticular part of internal capsule (L:3)/Posterior corona radiata (L:39)/Posterior thalamic radiation (L:3)/Tapatum (L:49)/	-27	-39	18	906.06	0.045	187
Inferior parietal lobule (L:15)/Supramarginal gyrus (L:3)/	Superior longitudinal fasciculus (L:5)/	-34.5	-45	33	902.28	0.046	72
None	Body of corpus callosum (3)/Splenium of corpus callosum (7)/	-16.5	-33	34.5	884.79	0.048	42

*Labelings of the anatomical regions of gray matter were based on the WFU PickAtlas Tool (http://www.fmri.wfubmc.edu/cms/software#PickAtlas/) [19, 20] and on the PickAtlas automated anatomical labeling atlas option [21]. Temporal pole areas included all subregions in the areas of this atlas.

**The anatomical labels and significant clusters of major white matter fibers were determined using the ICBM DTI-81 Atlas (http://www.loni.ucla.edu/).

,

Supplemental Table 4

Brain regions that exhibited significant negative correlations between sleep quality and MD

Included gray matter areas*(number of significant voxels in left and right side of each anatomical area)	Included large bundles** (number of significant voxels in left and right side of each anatomical area)	x	y	z	TFCE value	Corrected *p* value (FWE)	Cluster size (voxel)
Amygdala (R:3)/Angular gyrus (L:253)/Calcarine Cortex (L:198)/Caudate (R:303)/Anterior cingulum (L:641)/Middle cingulum (L:724, R:698)/Posterior cingulum (L:98, R:1)/Cuneus (L:93)/Inferior frontal operculum (L:329)/Inferior frontal orbital area (L:22)/Inferior frontal triangular (L:572)/Middle frontal orbital area (L:28)/Middle frontal other areas (L:1137)/Superior frontal medial area (L:366, R:62)/Superior frontal orbital area (L:4)/Superior frontal other areas (L:1053, R:451)/Fusiform gyrus	Genu of corpus callosum (383)/Body of corpus callosum (549)/Splenium of corpus callosum (585)/Cerebral peduncle (R:281)/Anterior limb of internal capsule (L:4, R:248)/Posterior limb of internal capsule (L:15, R:389)/Retrolenticular part of internal capsule (L:267, R:60)/Anterior corona radiata (L:532)/Superior corona	30	-21	-21	1514.34	0.007	35804

(L:158, R:514)/Heschl gyrus (L:13)/Hippocampus (L:46, R:459)/Insula (L:184, R:475)/Lingual gyrus (L:130)/Inferior occipital lobe (L:509)/Middle occipital lobe (L:998)/Superior occipital lobe (L:81)/Pallidum (R:413)/Paracentral lobule (L:376)/Parahippocampal gyrus (R:53)/Inferior parietal lobule (L:129)/Superior parietal lobule (L:288)/Postcentral gyrus (L:587, R:178)/Precentral gyrus (L:635, R:338)/Precuneus (L:277, R:18)/Putamen (R:466)/Rolandic operculum (L:30, R:127)/Supplemental motor area (L:293, R:523)/Supramarginal gyrus (L:136, R:13)/Inferior temporal gyrus (L:898, R:181)/Middle temporal gyrus (L:872, R:18)/Superior temporal gyrus (L:20, R:65)/Thalamus (R:111)/Thalamus (L:10, R:3)/	radiata (L:1255, R:1033)/Posterior corona radiata (L:534, R:765)/Posterior thalamic radiation (L:696, R:57)/Sagittal stratum (L:250, R:504)/External capsule (L:76, R:469)/Cingulum (L:347, R:223)/Stria terminalis (L:4, R:212)/Superior longitudinal fasciculus (L:452, R:618)/Superior fronto-occipital fasciculus (R:57)/Inferior fronto-occipital fasciculus (R:158)/Uncinate fasciculus (R:4)/Tapatum (L:70, R:13)/						
		-16.5	45	36	881.5	0.049	12
Superior frontal other areas (L:12)/	None				5		

Inferior temporal gyrus (L:2)/	None	-62	-42	-19.5	877.59	0.049	1
Cerebellum (L:1)/	None	-24	-88.5	-21	877.39	0.049	1
Anterior cingulum (R:2)/	None	10.5	43.5	4.5	874.54	0.050	2

*Labelings of the anatomical regions of gray matter were based on the WFU PickAtlas Tool (http://www.fmri.wfubmc.edu/cms/software#PickAtlas/) [19, 20] and on the PickAtlas automated anatomical labeling atlas option [21]. Temporal pole areas included all subregions in the areas of this atlas.

**The anatomical labels and significant clusters of major white matter fibers were determined using the ICBM DTI-81 Atlas (http://www.loni.ucla.edu/).

Supplemental Table 5. Associations between sleep length and each measure.

Measure	Best-fit model[a]	Adjusted R^2	*p* value of the best-fit model
POMS[b]–Tension–Anxiety	Linear, negative	-0.001	0.530
POMS–Depression–Dejection	Quadratic, positive	0.002	0.146
POMS–Anger–Hostility	Linear, negative	-0.001	0.783
POMS–Vigor–Activity	Linear, positive	$-2.30*10^{-4}$	0.394
POMS–Fatigue–Inertia	Linear, negative	0.004	0.013
POMS–Confusion–Bewilderment	Quadratic, positive	0.002	0.135
TCI[c]-Novelty Seeking	Quadratic, positive	0.008	0.004
TCI-Harm Avoidance	Linear, negative	-0.001	0.925
TCI-Reward Dependence	Quadratic, negative	0.006	0.010
TCI-Persistence	Quadratic, negative	0.014	$7.05*10^{-5}$
TCI-Self Directedness	Quadratic, negative	0.005	0.023
TCI-Cooperativeness	Quadratic, negative	0.009	0.001

TCI-Self Transcendence	Linear, negative	0.005	0.007
RAPM[d]	Linear, positive	-0.001	0.683
Stroop interference	Linear, positive	0.008	0.001
S-A creativity test	Linear, positive	-0.001	0.553
TBIT[e] percepion factor	Linear, negative	$-6.01*10^{-5}$	0.336
MD in the left globus pallidus	Linear, positive	0.002	0.056
MD in the right globus pallidus	Linear, positive	0.002	0.052
MD in the left putamen	Linear, positive	0.005	0.008
MD in the right putamen	Quadratic, positive	0.004	0.032
MD in the left hippocampus	Linear, positive	$-2.75*10^{-4}$	0.413
MD in the right hippocampus	Linear, positive	$-1.61*10^{-4}$	0.369

[a]Best-fit model of the correlation between sleep length and each measure using the Akaike Information Criterion.

Supplemental Figure legends.

Supplemental Fig 1. Distribution of sleep duration and sleep quality in our sample.

Supplemental Fig 2. The positive correlation between sleep length and MD. Glass brain views show tendencies toward positive correlation between sleep length and MD. (a) The results without correcting the effects of rGMD and rCSFD at each voxel(the same design as the analyses of the main text). The results are shown at a threshold of $p < 0.001$, uncorrected. (b) The results correcting the effects of rGMD and rCSFD at each voxel. The results are shown with a threshold of $p < 0.001$, uncorrected. Similar tendencies are obtained regardless of whether the effects of rGMD and rCSFD are corrected at each voxel.

(c) The results without correcting the effects of rGMD and rCSFD at each voxel (the same design as the analyses of the main text). The results are shown with a threshold of $P < 0.05$, corrected for FDR. (d) The results correcting the effects of rGMD and rCSFD at each voxel. The results are shown with a threshold of $p < 0.05$, corrected for FDR. Similar significant results are obtained regardless of whether the effects of rGMD and rCSFD are corrected at each voxel.

Supplemental Fig 3. The negative correlation between sleep quality and MD. Glass brain views show the tendencies toward negative correlation between sleep quality and MD. (a) The results without correcting the effects of rGMD and rCSFD at each voxel(the same

design as the analyses of the main text). The results are shown with a threshold of $p < 0.001$, uncorrected. (b) The results correcting the effects of rGMD and rCSFD at each voxel. The results are shown with a threshold of $p < 0.001$, uncorrected. Similar tendencies are obtained regardless of whether the effects of rGMD and rCSFD at each voxel are corrected.

Supplemental Fig 1.

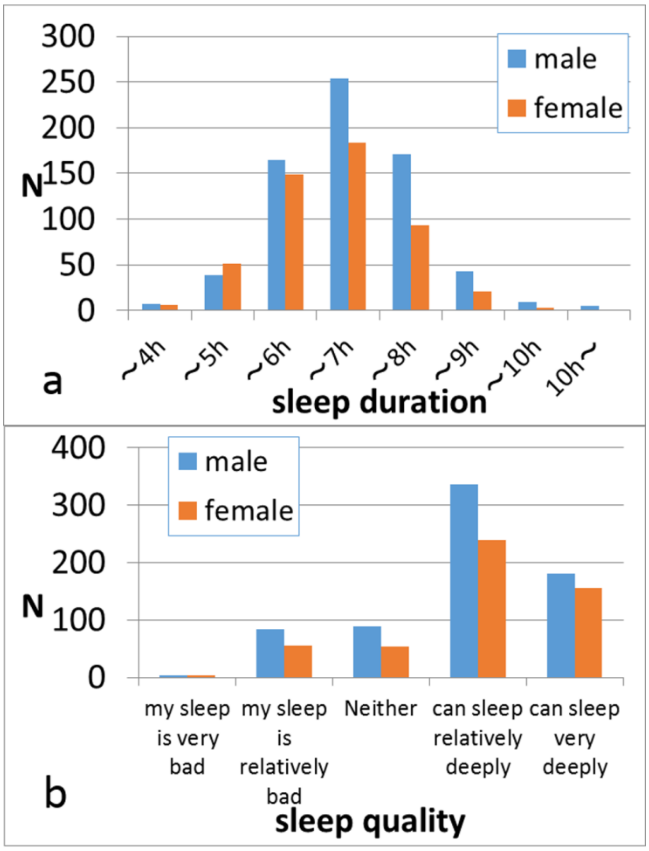

Supplemental Fig 2.

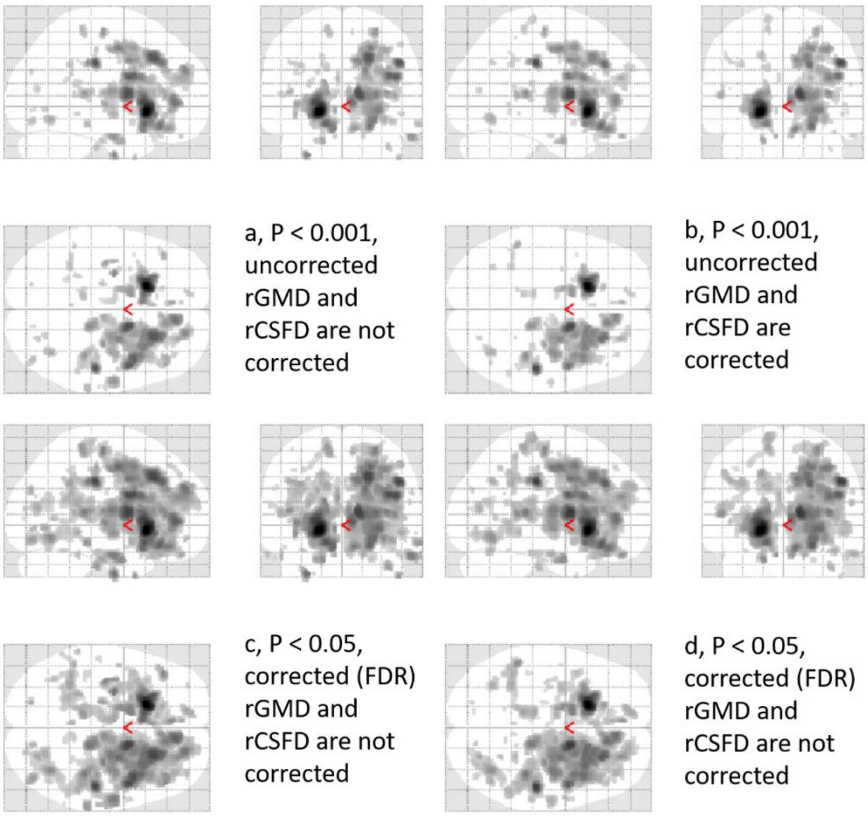

Supplemental Fig 3.

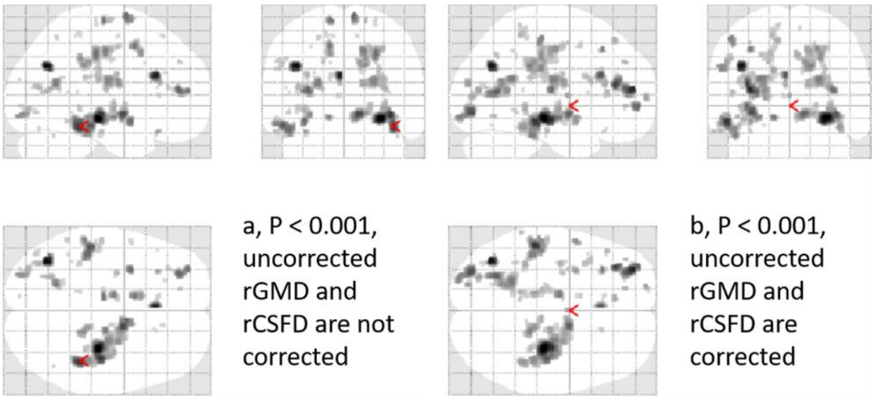

a, $P < 0.001$, uncorrected rGMD and rCSFD are not corrected

b, $P < 0.001$, uncorrected rGMD and rCSFD are corrected

References

1. Takeuchi, H. et al. Degree centrality and fractional amplitude of low-frequency oscillations associated with Stroop interference. *Neuroimage* **119**, 197-209 (2015).
2. Takeuchi, H. et al. Effects of Multitasking-Training on Gray Matter Structure and Resting State Neural Mechanisms. *Hum. Brain Mapp.* **35**, 3646-3660 (2014).
3. Oldfield, R.C. The assessment and analysis of handedness: the Edinburgh inventory. *Neuropsychologia* **9**, 97-113 (1971).
4. Takeuchi, H. et al. White matter structures associated with emotional intelligence: Evidence from diffusion tensor imaging. *Hum. Brain Mapp.* **34**, 1025-1034 (2013).
5. Takeuchi, H. et al. White matter structures associated with empathizing and systemizing in young adults. *Neuroimage* **77**, 222-236 (2013).
6. Takeuchi, H. et al. Training of Working Memory Impacts Structural Connectivity. *J. Neurosci.* **30**, 3297-3303 (2010).
7. Takeuchi, H. et al. White matter structures associated with creativity: Evidence from diffusion tensor imaging. *Neuroimage* **51**, 11-18 (2010).
8. Takeuchi, H. et al. Verbal working memory performance correlates with regional white matter structures in the fronto-parietal regions. *Neuropsychologia* **49**, 3466-3473 (2011).
9. Taki, Y. et al. Linear and curvilinear correlations of brain white matter volume, fractional anisotropy, and mean diffusivity with age using voxel-based and region of interest analyses in 246 healthy children. *Hum. Brain Mapp.* **34**, 1842-1856 (2013).
10. Barnea-Goraly, N. et al. White matter development during childhood and

adolescence: a cross-sectional diffusion tensor imaging study. *Cereb. Cortex* **15**, 1848-1854 (2005).

11. Le Bihan, D. et al. Diffusion tensor imaging: concepts and applications. *Journal of Magnetic Resonance Imaging* **13**, 534-546 (2001).
12. Takeuchi, H. et al. Impact of videogame play on the brain's microstructural properties: Cross-sectional and longitudinal analyses. *Mol. Psychiatry* **21**, 1781-1789 (2016).
13. Casanova, R. et al. Biological parametric mapping: a statistical toolbox for multimodality brain image analysis. *Neuroimage* **34**, 137-143 (2007).
14. Hashimoto, T. et al. Neuroanatomical correlates of the sense of control: Gray and white matter volumes associated with an internal locus of control. *Neuroimage* **119**, 146-151 (2015).
15. Jung, R.E. et al. Neuroanatomy of creativity. *Hum. Brain Mapp.* **31**, 398-409 (2010).
16. Dewald, J.F., Meijer, A.M., Oort, F.J., Kerkhof, G.A. & Bögels, S.M. The influence of sleep quality, sleep duration and sleepiness on school performance in children and adolescents: a meta-analytic review. *Sleep medicine reviews* **14**, 179-189 (2010).
17. Vincent, N., Cox, B. & Clara, I. Are personality dimensions associated with sleep length in a large nationally representative sample? *Compr. Psychiatry* **50**, 158-163 (2009).
18. Shimoyama, H. A STUDY ON THE SUBCLASSIFICATION OF MORATORIUM OF UNIVERSITY STUDENTS:relation to the identity development. *The Japanese Journal of Educational Psychology* **40**, 121-129

(1992).

19. Maldjian, J.A., Laurienti, P.J. & Burdette, J.H. Precentral gyrus discrepancy in electronic versions of the Talairach atlas. *Neuroimage* **21**, 450-455 (2004).

20. Maldjian, J.A., Laurienti, P.J., Kraft, R.A. & Burdette, J.H. An automated method for neuroanatomic and cytoarchitectonic atlas-based interrogation of fMRI data sets. *Neuroimage* **19**, 1233-1239 (2003).

21. Tzourio-Mazoyer, N. et al. Automated anatomical labeling of activations in SPM using a macroscopic anatomical parcellation of the MNI MRI single-subject brain. *Neuroimage* **15**, 273-289 (2002).
